# A comparison between a maximum care university hospital and an outpatient clinic – potential for optimization in arthroscopic workflows?

**DOI:** 10.1186/s12913-023-10259-3

**Published:** 2023-11-28

**Authors:** Martin Schenk, Juliane Neumann, Nadine Adler, Tilo Trommer, Jan Theopold, Thomas Neumuth, Pierre Hepp

**Affiliations:** 1 Innovation Center Computer Assisted Surgery (ICCAS), Leipzig University, Semmelweisstr. 14, 04103 Leipzig, Germany; 2 Department of Orthopaedic, Trauma and Plastic Surgery, Division of Arthroscopic Surgery and Sports Medicine, University of Leipzig Medical Center, Leipzig, Germany; 3 Sportklinik.ERFURT, Erfurt, Germany

**Keywords:** Surgical workflow analysis, Arthroscopy, Surgical process optimization, Operating room management

## Abstract

**Background:**

Due to the growing economic pressure, there is an increasing interest in the optimization of operational processes within surgical operating rooms (ORs). Surgical departments are frequently dealing with limited resources, complex processes with unexpected events as well as constantly changing conditions. In order to use available resources efficiently, existing workflows and processes have to be analyzed and optimized continuously. Structural and procedural changes without prior data-driven analyses may impair the performance of the OR team and the overall efficiency of the department. The aim of this study is to develop an adaptable software toolset for surgical workflow analysis and perioperative process optimization in arthroscopic surgery.

**Methods:**

In this study, the perioperative processes of arthroscopic interventions have been recorded and analyzed subsequently. A total of 53 arthroscopic operations were recorded at a maximum care university hospital (UH) and 66 arthroscopic operations were acquired at a special outpatient clinic (OC). The recording includes regular perioperative processes (i.a. patient positioning, skin incision, application of wound dressing) and disruptive influences on these processes (e.g. telephone calls, missing or defective instruments, etc.). For this purpose, a software tool was developed (‘s.w.an Suite Arthroscopic toolset’). Based on the data obtained, the processes of the maximum care provider and the special outpatient clinic have been analyzed in terms of performance measures (e.g. Closure-To-Incision-Time), efficiency (e.g. activity duration, OR resource utilization) as well as intra-process disturbances and then compared to one another.

**Results:**

Despite many similar processes, the results revealed considerable differences in performance indices. The OC required significantly less time than UH for surgical preoperative (UH: 30:47 min, OC: 26:01 min) and postoperative phase (UH: 15:04 min, OC: 9:56 min) as well as changeover time (UH: 32:33 min, OC: 6:02 min). In addition, these phases result in the Closure-to-Incision-Time, which lasted longer at the UH (UH: 80:01 min, OC: 41:12 min).

**Conclusion:**

The perioperative process organization, team collaboration, and the avoidance of disruptive factors had a considerable influence on the progress of the surgeries. Furthermore, differences in terms of staffing and spatial capacities could be identified. Based on the acquired process data (such as the duration for different surgical steps or the number of interfering events) and the comparison of different arthroscopic departments, approaches for perioperative process optimization to decrease the time of work steps and reduce disruptive influences were identified.

**Supplementary Information:**

The online version contains supplementary material available at 10.1186/s12913-023-10259-3.

## Background

Arthroscopic interventions play an important role in the treatment of joint pathologies [1]. Due to the lower soft tissue trauma and the associated higher patient comfort [2] as well as the lower postoperative pain compared to open joint surgeries [3, 4], the number of arthroscopic interventions has increased significantly in the last decades [5]. Between 2015 and 2020, about 750.000 arthroscopic interventions were performed per year, representing approximately 4.65% of all inpatient surgeries in Germany [6]. In 2020, for example, arthroscopic surgeries on knee articular cartilage and menisci, arthroscopic refixation and plastic surgeries on the capsular ligament apparatus of the shoulder joint, and arthroscopic surgeries on the synovium were among the top 20 most frequently performed full inpatient operations in Germany [7]. Arthroscopic interventions, in general, were in third place of all inpatient procedures [8].

In addition to inpatient arthroscopies, numerous procedures are performed in the outpatient sector. Reasons include advantages within the supply chain, standardized workflows, bypassing nosocomial infections, and a lower risk of thrombosis [2]. Whether an arthroscopic intervention is performed in an inpatient or outpatient clinic depends on various factors, such as the patient's general condition or the type of intervention. A continuously increasing number of outpatient arthroscopies is expected in the future [3]. Therefore, the improvement in the process and treatment quality of arthroscopic surgeries as well as the economic success is of special interest for maximum care providers but also for orthopedic outpatient clinics.

The surgical department is the most cost-intensive functional unit of a hospital [9–12]. Due to economic pressure and demographic developments [13, 14], hospitals are therefore endeavoring to optimize existing processes [15, 16] in order to utilize OR resources and capacities efficiently. Process analysis, optimization, digitization, and standardization [17] can lead to more efficient workflows and higher capacity utilization, but also improvements in terms of working atmosphere and employee satisfaction [9, 16]. Although, every department, OR, and surgical procedure has its unique processes with unique problems, different commonly occurring influencing factors were identified in the literature, e.g. the OR's daily planning, the punctuality of the individual actuators, the organization between the procedures (OR preparation, patient preparation, setup, etc.), distractions during the procedure, and the collaboration of the OR team [9, 10, 14, 16, 18–22]. Perioperative processes are highly complex, intertwined with significant influence on each other, and have important implications on related clinical processes, patient outcome, and safety. Even small delays can lead to timing issues that affect the entire surgical OR team and the overall OR performance [23, 24]. Surgical processes are highly variable and individual to the patient, the OR team, available resources as well as hospital processes and standards. This complexity requires a comprehensive data-driven surgical workflow analysis from temporal, behavioral, structural, and operational perspectives before restructuring or implementing new processes in the surgical department. A data-driven process analysis is based on facts, metrics, and data in order to objectivize the decision-making for process optimization. Additionally, it provides a deeper understanding of the decisions’ impact prior to the implementation of process optimization measures.

The aim of this work is to perform such a data-driven process analysis and subsequently optimization for arthroscopic surgery with a newly developed software toolset^1^ in a maximum care university hospital (UH) and a high-throughput specialty joint surgery (OC) outpatient clinic. With this approach, it is possible to record and analyze perioperative processes in order to gain a better knowledge of arthroscopic processes. In this way, commonalities and differences of the arthroscopic interventions at the OC and UH can be identified and eventually, optimization approaches can be deduced to improve the performance of perioperative processes.

In this study, the following research questions will be addressed using the data-driven surgical workflow analysis: The OC performs significantly more arthroscopic interventions per year than the UH (OC ~ 3400; UH: ~ 400). But does the number of cases have an impact on the processes and the standardization of workflows? What influence do other factors have, such as the spatial requirements or the personnel composition? And which perioperative optimization potentials can be deduced from the surgical workflow analysis for OC and UH and arthroscopic procedures in general?

To answer these questions a database is created from the recorded interventions at the OC and the UH, such as the sequence and duration of perioperative phases and individual work steps or the frequency of disruptive influences. Following a detailed workflow analysis, realistic and feasible optimization potentials have been identified on the basis of the data obtained. To the best of our knowledge, this is the first approach for a comprehensive data-driven surgical workflow analysis in the domain of arthroscopic surgery, which includes general recommendations for process optimization and a software toolset for further individual process analysis.

### State of the art

The understanding of surgical processes requires the gathering of specific and valuable information from various perspectives. Process acquisition is the prerequisite for analyzing existing processes and generating optimization approaches and improved target processes. To achieve a complete surgical process representation, intraoperative (live) workflow recordings of a human observer [25], which is used in this study, or video annotations [26] can be used. With the recorded process information, surgical interventions can be described with different levels of granularity. The surgical procedure consists of phases (e.g. preparation, procedure, and follow-up), which form an overall process that is divided into complex sub-processes. These sub-processes consist of a sequence of activities, which however can be represented with time parameters and a combination of the *actuator*, the *surgical action*, the treated *anatomical structure*, and the used *instruments/resources* (e.g. the *surgeon cuts* the *skin* with a *scalpel*) [25].

The live acquisition of surgical processes is usually achieved with the help of a software-based workflow recording tool. In this work, the adaptable surgical workflow recorder *s.w.an Suite* (in-house development [25]) was used and extended by further analysis tools for arthroscopic interventions. With the s.w.an Suite, workflows from ENT, neurosurgery, cardiac surgery, ophthalmic surgery, and interventional radiology have been recorded in numerous disciplines, interventions, and different applications. The data obtained can then form the basis of *Surgical Process Models (SPMs)* [27]. SPMs represent the temporal course of sequential and parallel phases, actions, and events [28] and can be used to describe whole surgical procedures. Furthermore, SPMs provide a deeper understanding of the processes involved [29] and reveal optimization potential [24, 30]. Based on SPMs, process mining techniques, such as filtering, highlighted visualization, process comparison, deviation, and conformance analysis as well as performance, bottleneck, and root cause analysis can be performed. In addition, SPMs can be executed in simulation scenarios and assessed based on defined key performance indicators. In this way, the best process alternative can be determined regarding the desired optimization goal (e.g. improvement in OR utilization or reduction of process duration). In literature, surgical workflow analyses have been applied e.g. to the comparison of intervention populations [27], surgical skill analysis [31–33], OR optimization [34–36], and the development of surgical assistance systems [25, 37]. The subsequent implementation of optimization approaches can lead to more efficient workflows within the individual surgical processes and thus significantly influence the overall process in the OR department.

## Material and methods

### Data acquisition

**Fig. 1:**
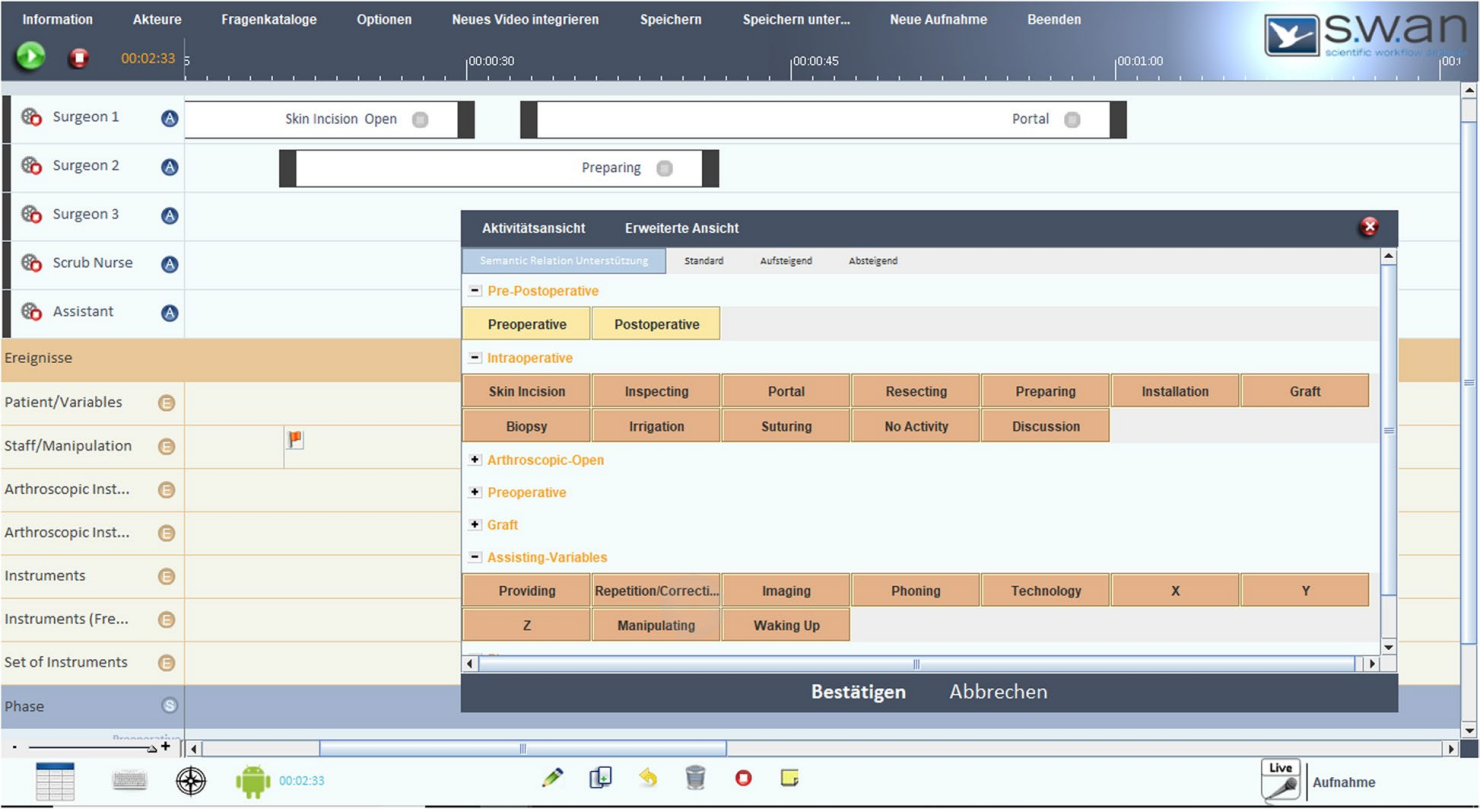
Recording of surgical workflows with the s.w.an Suite

The data acquisition of the arthroscopic interventions was conducted utilizing the s.w.an Suite [25]. The software is an in-house development, which was developed specifically for the recording of surgical workflows. Based on the software, a specialized surgical workflow recording, analysis, and perioperative process optimization toolset for arthroscopic surgeries was developed. For workflow acquisition, the s.w.an Suite surgical workflow editor needs to be configured for the required surgical intervention type. For this purpose, different arthroscopic interventions were observed without the software to identify possible activities and workflows, anatomical structures, used instruments as well as disturbing influences, repetitions, and bottlenecks of the processes. Based on the acquired findings, the surgical workflow editor was configured to enable the recording and representation of pre-, post-, and intraoperative processes in different arthroscopic procedures (Fig. 1). The records were captured live in the operating theater. First, some interventions were recorded by two observers. Since there was no difference in quality with regard to the completeness and consistency of the data, the following interventions were recorded by one observer and further analyzed postoperatively [38].

In addition to the recording of preoperative and postoperative activities and the intraoperative surgical workflow, a special focus was on the identification of distractions and disturbing influences on the processes. Disruptive influences and deviations were defined as events and actions that delay surgery (for example, the absence of a needed instrument or defective devices). Distractions are associated with poorer team performance [39], may compromise surgical quality and patient safety [40], and have a considerable influence on the temporal progress of the intervention.

#### Recording of the arthroscopic interventions

**Fig. 2:**
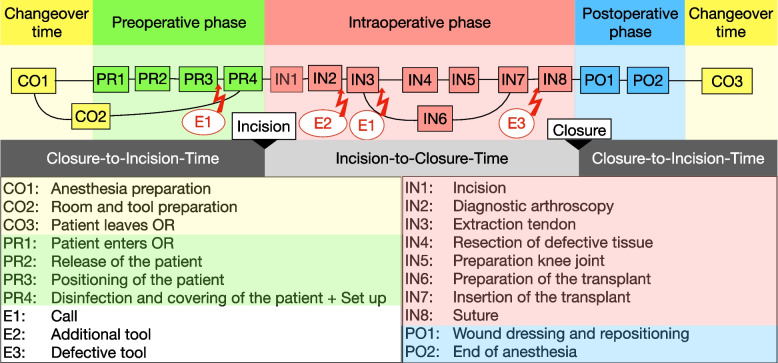
High-level gSPM of the perioperative process in arthroscopic surgeries (example of anterior cruciate ligament reconstruction (ACL) surgeries)

The recording of perioperative processes is composed of the acquisition of surgical phases and surgical activities, actuators performing the activities, and resources, which are used to perform the activities. The phases, which are shown in Fig. 2, constitute the temporal frame for the different sub-processes and actions and define the current subsection of the intervention. The phases of the surgery were labeled as “changeover time” (time between two consecutive operations when no patient is in the OR), “preoperative”, “intraoperative” (or “Incision-to-Closure-time”), and “postoperative”. According to [41], “*Incision-to-Closure-time*” (ICT) is defined as the period between the beginning of the incision of the skin and the end of the final surgical suture. Based on the recording of substantial surgical phases OR key performance indicators (KPI), such as the “*Closure-to-Incision-time*” (CIT) or the “*Anesthesia Ready-to-Incision-Time*” were calculated. The CIT is the period between the end of the closure of a surgical session and the beginning of the incision of the following session [42]. The phases are recorded (e.g. pre- or intraoperative phase) or calculated during analysis (e.g. CIT). The definitions of the phases and OR KPI correspond to the conventional specifications from the literature [41] and are also presented in the Additional file 1.

In addition to the phases, the sub-processes of the phases were considered. These are divided into actions and events. Actions are work steps with a duration (time course), which are performed by an actuator Events are a point in time, and occurrences are documented as a single time stamp. (The following scenario should serve as an example: During the intervention, an instrument is missing and is supplied by an assistant. In this case, the missing instrument is the event and the subsequent provision of the instrument is the action, which duration is measured). Therefore, actions and events must be analyzed together. For the recording of actions, the start and end time of every work step is logged in detail with regard to potential pauses and parallel actions. Thereby, it is possible to record different actuators (e.g. surgeons, scrub nurses) and allocate one or more actions to the actuators (Fig. 1).

Due to the high number of different actions, the focus was placed on processes that have an impact on the time course and can potentially delay the operation. Standardized actions (for example, positioning the patient and applying the wound dressing) were distinguished from unplanned actions (for example, making phone calls and providing additional material). Verbal interactions were recorded as events since the actual actions were not interrupted. Actions resulting from a verbal interaction (e.g., it is discussed in the consultation that an instrument needs to be provided) were documented. Audio recordings of the conversations were not made. When recording intraoperative actions performed by surgeons, it is important to keep in mind that UH is a teaching hospital. Therefore, the focus was placed primarily on recording actions in the preoperative and postoperative phases as well as in the changeover time.

Regarding the actuators and their actions, it is apparent which person performs an action in a respective phase of the intervention, and how much time the action requires. Furthermore, general information about the procedure, the time, and the number of occurrences of events (e.g. disruptions, the arrival of the patient and personnel in the OR, etc.) can be documented with the s.w.an Suite. Thereby, special attention was paid to events that may have an impact on the timing of operations (e.g. additional and defective tools, opening of additional instrument sets, incoming and outgoing phone calls, and provision of materials and supplies). Some of these events can also trigger actions (for example, the event 'additional instrument' is followed by the action 'Provisioning within the OR'). Additionally, a questionnaire was created that covers general and special information about the procedure.

In this way, 53 arthroscopic interventions on 26 observation days were recorded at the UH. At the OC 66 arthroscopic interventions were recorded (Table 1). Interventions that were entirely arthroscopic or combined arthroscopic and open processes were considered. The processes were recorded by three trained observers with medical background. The recording person was in the operating room during the entire admission period in order to record the perioperative processes.

**Table 1:** Number and type of recorded arthroscopic interventions at UH and OC

Joint	UH n (%)	OC n (%)	Total n (%)
Knee	26 (49.06)	53 (80.30)	79 (66.39)
Shoulder	17 (32.08)	12 (18.18)	29 (24.37)
Ankle joint	4 (7.55)	1 (1.52)	5 (4.20)
Hip	3 (5.66)	0 (0)	3 (2.52)
Elbow	3 (5.66)	0 (0)	3 (2.52)
*All*	*53 (100)*	*66 (100)*	*119 (100)*

### Data processing

Based on the recorded data, the clinic's internal processes have been analyzed in detail. Therefore, every recorded intervention was saved as a structured XML file, which enables straightforward postprocessing, statistical analysis, and subsequent in-depth process analysis. For an initial overview of the perioperative processes, the s.w.an Suite provides a workflow analytics tool. The tool analyzes for example the duration of surgical phases (preoperative, postoperative, etc.), the number and duration of activities, and the number and type of (disrupting) events. In addition, a basic process visualization of the perioperative workflow is presented.

Subsequently, a more detailed process analysis was performed, to identify process parts with potential for optimization. For this purpose, the recorded workflow data (XML files of the interventions) were transferred to an SQL-Database with the help of the s.w.an Suite Arthroscopic Toolset. In this way, complex SQL queries can be used to search, filter, and group the workflow data (e.g. provide a list of events, where a defective instrument occurred during the intraoperative phase in all recorded arthroscopic knee surgeries). Furthermore, the workflow data can also be imported directly into process mining tools for a more in-depth analysis, which targets the identification of activities with prolonged durations as well as process bottlenecks, or possible parallelization strategies.

#### Statistical analysis

The basic process analysis included a comparison of perioperative process times and key performance indicators between the UH and OC. The evaluation includes the analysis of the phases, actions, and occurrences during the perioperative process in connection with the observation of the circumstances. The statistical analysis of the perioperative processes was performed using SQL queries and spreadsheet software (Microsoft Excel and Apple Numbers) subsequently. Data were summarized as mean with standard deviation (SD). Parametric paired tests (t-Test, two-sided, same sample) were used for the comparison of the two clinics. The level of significance was defined as *p* < 0.05.

### Workflow analysis and process mining

Due to the complexity of the arthroscopic indications and the high granularity of the data, the recorded interventions were considered both as a whole and according to specific criteria, such as the type of joint or the type of surgery. The processes were modeled as Surgical Process Models (SPMs). “SPMS can be defined as simplified, formal, or semiformal representations of a network of surgery-related activities, reflecting a predefined sub-set of interest [43].” SPMs are mathematical models that represent the process of an intervention type. In literature, example implementations are described using Hidden Markov models [37], Random Forests [44], or Workflow Nets [45]. The models can be used to perform computations and predictions [46]. For the current study, SPMs were used to perform a detailed analysis of the perioperative processes and to identify optimization potentials.

SPMs provide an improved understanding of the logical and temporal course of an intervention as well as influencing factors and performance indicators. A distinction is made between individual surgical process models (iSPMs), which represent a single procedure, and generic surgical process models (gSPMs). The gSPM is a model that is calculated from the individual iSPMs and thus represents the average overall process. For example, there were computed gSPMs using the data of all arthroscopic interventions, all arthroscopic knee interventions, or all knee interventions with cruciate ligament surgery, which is presented in Fig. 2 as an example of a high-level gSPM). Grouped in the phases, common perioperative activities of arthroscopic surgeries, potential disturbances, and disrupting events (e.g. phone calls, additional instruments, etc.) are depicted. The iSPMs and gSPMs enable the comparison between single and average processes in and between the clinics. The models were generated also using the s.w.an Suite Arthroscopy Toolset. In addition, process mining techniques were utilized to identify exceptionally long activities in the workflow or long interval times between consecutive process steps. For process mining, the software tools PROM [47] and Disco [48] were used.

Another aspect of the data analysis was to examine roles, tasks, and responsibilities within the surgical team. For this purpose, the respective executing actuators and the cooperation in the OR team were also recorded and analyzed in addition to the intraoperative processes. Furthermore, the spatial and personnel requirements and resources of the two clinics were analyzed and compared. Due to the considerable influence of disruptions on surgical performance, distracting factors were recorded, and a root-cause analysis was performed. Based on the process analysis, process optimization potentials have been identified and corrective actions for disrupting influences on the perioperative workflow have been proposed.

### Comparison of the clinics

The clinics involved in this study were chosen deliberately because fundamentally different conditions prevail. At UH, an average of ~ 400 arthroscopic interventions are performed per year, compared to ~ 3400 at OC. While the UH is an academic teaching hospital, the OC specializes in joint pathologies and their treatment. Differences in terms of the prerequisites have been identified. While at the OC three ORs can be used in parallel every day for joint surgeries (including a large number of arthroscopies), at the UH only one OR is available for arthroscopic interventions on three days per week. Similar materials and instruments were used at both clinics. It was noticeable that at the OC special arthroscopic draping solutions were used.

At the OC, there is an anesthesiologic preparation area in front of the ORs. The completion of the anesthesiologic induction then continues in the OR itself. After the surgery, the patients are transferred back to the preparation area upon awakening. At the UH, anesthesiologic preparation is performed in a preparation room next to the OR. The anesthesiologic release continues in the OR. After surgery, patients are awakening in the OR and then are transferred through a room next to the preparation room into the awakening area. The preparation area at OC is significantly more spacious than the preparation room at OC. At the OC, patients were positioned in a standardized procedure by the assistants. Deviations from the standards could only be identified in the case of special requirements of the surgeons. At UH, no SOPs existed regarding patient positioning.

The spatial and logistical requirements of OC offer optimal conditions for arthroscopic interventions, while at UH the OR is designed for a wide range of operations. Due to the specialization on arthroscopic interventions, higher case numbers exist at the OC, which is noticeable in the standardization of the surgical processes. The two clinics also differ significantly in terms of personnel requirements. At the UH, the entire surgical team has to cover a wide range of surgical interventions. Furthermore, there are changes within the assisting surgical team. The reason for this is the rotation and shift principle at the UH. In addition to changing scrub nurses, the surgeon team is also found in a changing composition and heterogeneous level of training within the framework of the teaching assignment. A constant and therefore very well-rehearsed team was established at the OC, which is specialized in joint surgery. Only specialized and experienced surgeons are involved in the intervention, as there are no regular teaching activities at the OC. At UH, however, surgeons have different levels of experience. Here, inexperienced surgeons will be trained by experienced surgeons. The assisting staff is also specialized in joint surgery. In contrast to the UH, the composition of the surgical team is characterized by significantly fewer changes not only in general but also during the course of the day. Additionally, the working day ends after the end of the last intervention and is therefore not tied to fixed times. The surgeons at the OC are mostly present in the OR department throughout the day, while the UH surgeons are usually also active in other areas of the clinic and are only called in at certain times in the surgical process. In general, there are significantly more persons involved at the UH (2–3 surgeons, 1 anesthesiologist, 3–4 scrub nurses) than at the OC (1 surgeon, 1 anesthesiologist, 3–4 scrub nurses).

## Results

### Analysis and comparison of perioperative phases

**Fig. 3:**
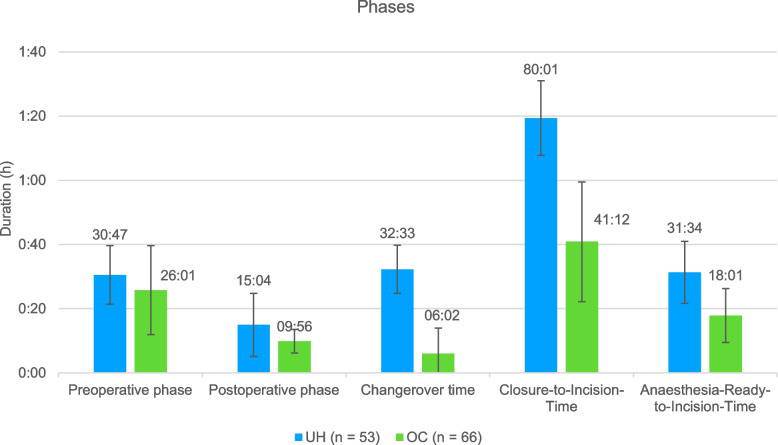
Duration of phases at UH (blue) and OC (green)

In Fig. 3, the pre-, and postoperative phases as well as the changeover time between two surgeries are presented for the UH and the OC. The preoperative phase lasted an average of 30:47 min (± 9:11 min) at UH and 26:01 min at the OC (± 13:59 min) which is a significant difference of 4:46 min (*p* < 0.05). With a difference of 5:08 min at UH (15:04 min, ± 9:55 min), the postoperative phase also lasted significantly longer (*p* < 0.001) than at the OC (9:56 min, ± 3:42 min). The changeover time at UH (32:33 min ± 7:35 min) lasted significantly longer (*p* < 0.001) than at the OC (6:02 min ± 8:03 min). With 18:01 min (± 8:26 min), the A*nesthesia Ready-to-Incision-Time* of the OC is also 13:33 min and thus significantly shorter (*p* < 0.001) than at the UH (31:34 min ± 9:44 min). At 80:01 min (± 11.43 min) the CIT was significantly longer (38:49 min, *p* < 0.001) at the UH than at the OC (41:12 min, ± 18:47 min).

### Analysis and comparison of actions

**Fig. 4:**
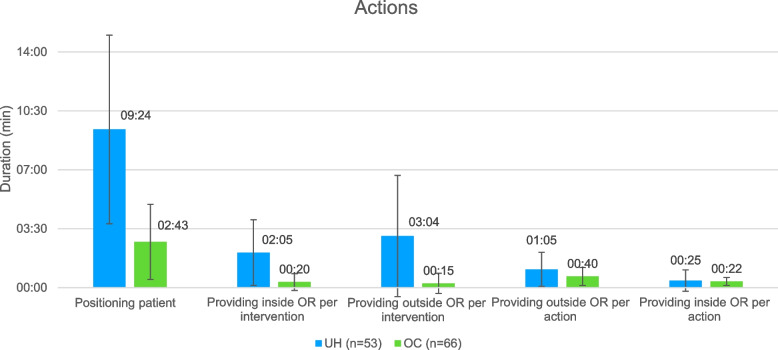
Duration of actions per surgery at UH (blue) and OC (green)

In Fig. 4 the actions per surgery are presented for both clinics. At UH, standardized perioperative actions such as patient positioning took longer than at OC (UH: 9:23 min ± 5:36 min, OC: 2:44 min ± 2:14 min, *p* < 0.001).

Furthermore, the OC required less time for resource and material provisions inside (UH: 2:05 min ± 1:57 min, OC: 0:20 min ± 0:30 min, *p* < 0.001), and outside of the OR (UH: 3:04 min ± 3:36 min, OC: 0:15 min ± 0:36 min, *p* < 0.001) per intervention. Additionally, the UH required more time for provisions inside (UH: 0:25 min, ± 0:38 min, OC: 0:22 min ± 0:14 min, *p* = 0.6), and outside the OR (UH: 1:05 min ± 1:01 min, OC: 0:40 min ± 0:32 min, *p* < 0.05) per action.

### Analysis and comparison of events

**Fig. 5:**
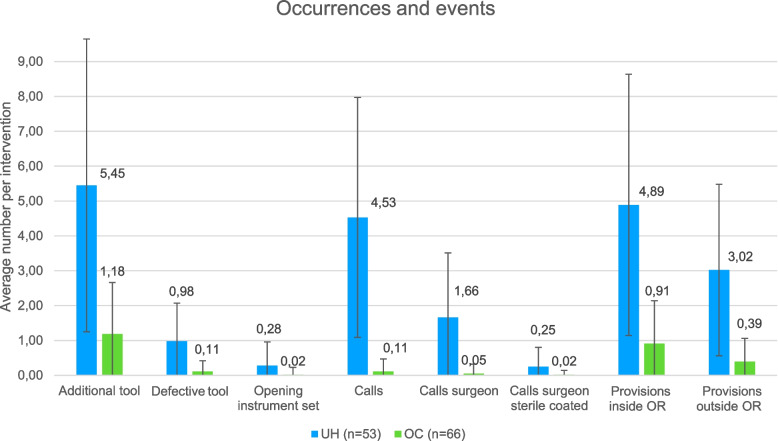
Occurrences of disruptive events per surgery at UH (blue) and OC (green)

In Fig. 5 the occurrences of disruptive events per surgery are depicted for UH and OC. At the UH there were an average of 5.45 (± 4.2) additional needed tools per procedure, while at the OC there were an average of 1.18 (± 1.48) additional tools (*p* < 0.001). Defective tools (UH: 0.98 ± 1.09; OC: 0.11 ± 0.31) were found significantly more (*p* < 0.001) at the UH. At the UH, an average of 4.89 (± 3.75) provisions were made inside and 3.02 (± 2.46) outside the OR. At the OC, 0.91 (± 1.48) provisions were made inside and 0.39 (± 0.67) outside the OR. This means that significantly fewer provisions were made at the OC, both inside (*p* < 0.001) and outside (*p* < 0.001) the OR. At the UH, an average of 4.53 (± 3.44) telephone calls were made per intervention, of which 1.66 (± 1.85) calls were made by a surgeon. At the OC, 0.11 (± 0.31) telephone calls were made per surgery, thereof 0.05 (± 0.27) calls by the surgeon. Consequently, at the OC were fewer calls in total (*p* < 0.001) and by the surgeon (*p* < 0.001). Also, fewer calls were made by the sterile coated surgeon (UH: 0.25 ± 0.55, OC: 0.02 ± 0.12, *p* < 0.005) at the OC. At the UH, more instrument sets were opened while the surgery has already started (UH: 0.28 ± 0.68, OC: 0.02 ± 0.21, *p* < 0.001).

### Analysis and comparison of SPMs with process mining techniques

**Fig. 6:**
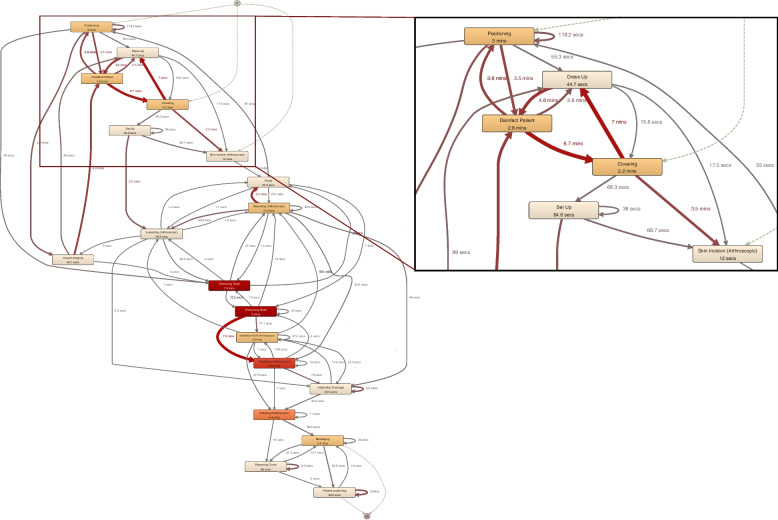
gSPM of anterior cruciate ligament reconstruction at OC (enlarged to the preoperative process)

Analyzing the different processes, overlaps, and similarities, as well as differences in various phases, have been identified with process mining analysis. At the OC, the preparation of the OR and the patient is mainly performed by the scrub nurses. The surgeon usually joins the team at a later stage. Thus, longer interval times occur between the preoperative actions (positioning, covering, disinfection, washing, dressing) (Fig. 6). The preparation is often completed by the time the surgeon arrives. The surgeon then intervenes in the preoperative actions for completion or if a correction is desired. At the UH, however, the preoperative phase is done in the presence of at least one surgeon who is involved in the preoperative actions. Two surgeons are usually involved in positioning the patient.

**Fig. 7:**
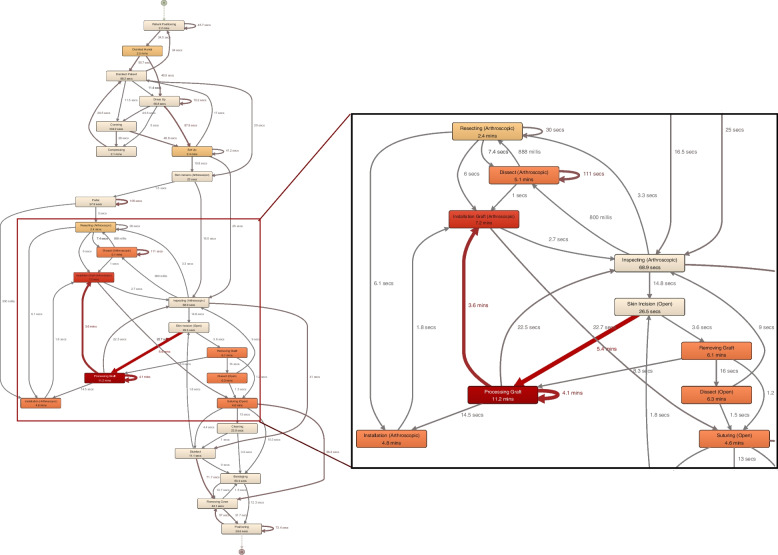
gSPM of anterior cruciate ligament reconstruction at UH

In Fig. 6, the gSPM of anterior cruciate ligament reconstruction (ACL) surgeries at OC is presented as an example of an in-depth analysis of intraoperative processes. The OC gSPM model contains 11 individual surgical process recordings merged into a generalized model with a colored representation of the activities' mean durations and the interval times between successive actions. Figure 7 shows the respective process model of the same surgery from the UH based on 10 individual surgical process recordings.

Also in the intraoperative phase, differences can be identified. At the UH, long interval times were recognized after the skin incision and the processing of the removed graft (Fig. 7). The surgery is performed by at least two surgeons. The main surgeon extracts the graft while the other one is assisting the process. Afterward, the assisting surgeon prepares the graft for installation. This parallel process fastens the removal process, but causes longer interval times in the generated gSPM, as on the one hand the assisting surgeon is on hold for the removal of the graft, and on the other hand, the main surgeon often waits for the finished preparation to install the graft into the patient. At the OC this task is performed by a scrub nurse, who is not directly assisting during surgery. Therefore, the OC needs more time for the removal of the corresponding transplant than the UH. In addition to the absence of an assisting surgeon, this circumstance is caused by the use of different types of transplants. While at the UH mainly the semitendinosus tendon is utilized, at the OC different tendons are used (semitendinosus gracilis, patellar tendon, quadriceps femoris tendon). As the UH is a teaching hospital, the assisting surgeon processing the graft is often a junior surgeon. This leads to delays in the reprocessing process, which accounts for a longer duration compared to the trained nurse at the OC.

## Discussion

In this multicentric study, differences and commonalities in perioperative workflows as well as process interferences, such as process bottlenecks, have been identified. Process bottlenecks are events and actions that have elementary relevance for the temporal course of an operation. These events and actions must be completed before the next processes can start. Examples include waiting for the surgeon to arrive, anesthetic preparation, or cleaning.

Based on the workflow analysis, common optimization potentials have been deduced for process redesign and process improvement. For this purpose, a software toolset for surgical workflow analysis and perioperative process optimization in arthroscopic surgery was developed. The toolset is based on an adaptable surgical workflow recording software [25] and provides functionalities for the acquisition, analysis, comparison, and optimization of arthroscopic surgeries at different demand-orientated levels of detail.

### Data acquisition

The s.w.an Suite has already been used in various surgical subjects and disciplines [25, 42, 49–52]. The configuration was based on live observations and to obtain relevant data. The recorded workflow data can be used to describe an individual surgical process (iSPM) and its sub-processes, a generic course of an intervention type (gSPM) as well as to identify sources of error and delays. Although the toolset is rather easy to use, possible influencing factors and limitations regarding the software and its application must be considered. The focus of the process analysis was not set on intraoperative activities. For example, the suture activity, which is part of the intraoperative phase, was performed (in part) by novices, who were supervised by experienced surgeons, which is common at teaching hospitals. Due to the high variability, different levels of experience, and different patient basis (inpatient and outpatient cases), a comparison of intraoperative activities and ICT between OC and UH is limited. Therefore, an analysis of intraoperative surgical performance (of a single surgeon or concerning the level of experience), as well as performance analysis of single persons of the OR team, were not performed. Therefore, the consideration of surgical subprocesses in this study is limited. For example, other studies had defined which action was performed with which surgeon's hand and instrument on a specific anatomical structure [27]. In this study, the focus was not set on high-granular intraoperative surgical activities, such as recording which hand is used by the surgeon on a specific anatomic structure. In order to temporally record all processes in the operating theater, events and actions were acquired in detail, in which more potential for process optimization was expected. Thus, the data granularity with respect to the surgical subprocesses is limited but reflects them sufficiently for this work. Furthermore, operations were recorded during live observations, which limited the detail of recorded events and actions. Studies on this topic show that it makes no significant difference whether the recordings were made live or from video footage [38]. The fact that the recordings were made by three different people also plays a minor role, especially since all the recorders were involved in the project and the configuration of the software [38]. Due to this, one can assume a constant data quality in order to analyze and compare the processes of clinics themselves and with each other.

### Workflow analysis and process mining

The spatial, process-related, and personal differences are noticeable, e. g. in the duration of patient positioning. Using similar positioning materials, the duration of the patient positioning was significantly shorter at the OC than at the UH.

At the OC, knee, shoulder, and ankle arthroscopies were recorded during the data acquisition period. Furthermore, it was noticeable that a high percentage of procedures were related to meniscus surgery. At the UH, on the other hand, in addition to knee, shoulder, and ankle arthroscopies, hip and elbow interventions were also recorded. The procedures themselves also differ to some extent between the two institutions. Certain differences could also be identified with regard to surgical technique. Taking anterior cruciate ligament surgery as an example, at the UH, the semitendinosus tendon is primarily used as a graft. At the OC, different replacement grafts (semitendinosus-gracilis-tendon, quadriceps-tendon, and patellar-tendon) were observed. The example of anterior cruciate ligament surgery also shows that the scrub nurses at the OC sometimes take on different intraoperative tasks than UH assistants during the operations, such as preparing the transplant or suturing the arthroscopic accesses. Furthermore, various perioperative actions (positioning, covering, wound dressing), which are performed by the surgeons at the UH, are carried out by the scrub nurses at the OC.

The duration of all phases at the OC is significantly shorter than at the UH. Evaluated actions that have an impact on the time course of the operation lasted longer at the UH. Moreover, fewer events occurred at the OC that influence the duration of the operation. The reasons for these results were primarily the spatial equipment, the organizational structure, as well as the team composition and experience of the nursing staff in dealing with arthroscopic interventions. If only recordings of arthroscopic operations performed by a well-rehearsed and experienced team were considered, comparable process times for preoperative and postoperative phases were achieved at UH compared to OC. The longer changeover times and CIT at UH are mainly related to the different spatial conditions and the available resources. At OC, several ORs are available, which can be prepared and post-processed in parallel, while a patient is receiving surgery at the same time. At the UH, one OR is available, thus sequential processing of the preparation and post-processing tasks must be performed. In return, more personnel resources are available at the UH to compensate for the spatial restrictions. Numerous studies have already shown that the parallel processing of preoperative and postoperative tasks has a positive effect on the overall duration of an operation [53–55]. On the other hand, the process mining evaluation of OC showed longer waiting times between the preoperative activities, which may be influenced by the parallel surgery approach. With the parallel method, longer waiting times often occur at the end of the preparation phase, as the patient is on hold for the arrival of the main surgeon.

Due to these prerequisites, the coordination, planning, and implementation of the interventions at the UH are more complex than at the OC. In addition to the challenge of data acquisition due to the different circumstances, the differences offered a starting point for optimization approaches to the analyzed processes. Due to the complexity and high amount of data in context with the prevailing conditions, numerous insights could be gained from process analysis. Since the focus was particularly on the main processes and sources of error, the data were primarily used to generate realistic optimization approaches. With regard to the results, significant differences between the two clinics were uncovered, which can be explained in part by the above-mentioned circumstances and should therefore be viewed critically. Nevertheless, commonalities could also be identified. When further examined from other perspectives, the data obtained also offers the potential for answering additional questions. The newly obtained SPMs can also contribute to a better understanding of surgical processes in the future and support them by integrating computer-based assistance systems.

### Process optimization recommendations

Perioperative processes are highly individual to the patient, the OR team, and the clinic. As different as the local conditions of an arthroscopic department are as many possibilities exist to improve the perioperative processes. Nevertheless, some general optimization potentials have been summarized in Table 2, which result from the present analysis, and literature review and have been proven effective in practice for optimizing surgical procedures. An important prerequisite for training inexperienced team members is clear structures regarding workflows in the operating room. SOPs can provide a good framework for the flow of standardized operations.

**Table 2:** Recommendations for process optimization in arthroscopic departments

Category	Optimization potential
Temporal and procedural factors	Punctual start of the surgery day - Fixed call times for all OR team members to be present in the OR (e.g. 1^st^ surgeon should be called in at the beginning of the anesthesiologic induction and 2^nd^ surgeon at the release by anesthesia)
	Definition of Standard Operating Procedures (SOPs) for common processes
	Parallelization of sequential processes, e.g - Parallel OR and patient preparation - Patient Positioning by the main surgeon and assistant to reduce potential positioning corrections - If possible in terms of spatial and personnel conditions a parallel anesthesia induction should be considered
	Optimization of OR planning and scheduling (e.g. [56, 57])
Behavioral and human factors	Implement a constant and well-rehearsed OR team experienced in arthroscopic interventions
	Identify a person responsible for calls (staff and patients)
	Minimize workflow disruptions, such as non-urgent telephone calls, especially in the intraoperative phase to ensure a quiet working atmosphere
	Identify persons responsible for the positioning of the patient
	Communication of OR planning to all members (type of surgery, preferred positioning, special needs, etc.)
	Dedicated cleaning and transport staff to one or more ORs
Operational and spatial factors	Fixed Positioning of the staff and needed resources (arthroscopy devices, OR table, C-arm, OR staff, etc.) - Positioning sketches should be prepared according to the type of procedure, the affected side, and needed resources to minimize ambiguity
	Minimizing needed resources (especially instrument sets) saves costs and time for preparation
	Implementation of fully prepared case carts for every surgery of the day
	Functionality tests of all OR devices and especially arthroscopic devices (arthroscope, camera, suction/irrigation devices, displays) in the preoperative phase to prevent delays from system failures
	Optimize material storage to reduce the time for material search
	Definition of material lists to prevent the provision of additional or missing materials and instruments during surgery
	Usage of arthroscopic draping solutions to collect irrigation fluid and minimize the postoperative cleaning effort and therefore change over time

The optimization of the perioperative processes and surgical interventions should be a permanently accompanying process that checks the perioperative workflows, evaluates them according to objective criteria, and continuously improves them. For this purpose, the s.w.an Suite arthroscopic toolset was developed, which enables the recording, analysis, and individual evaluation of the local conditions. The optimization potentials listed in Table 2 are intended to be a starting point for individual assessments of other arthroscopic departments.

In this study, two different approaches to OR utilization were described. At OC, there is a parallel approach in which two ORs are handled by one surgeon, while at UH there is one OR available, which implies a sequential preparation process. Whether a parallel or sequential approach is more suitable for an arthroscopic clinic depends mainly on the spatial and personnel conditions. An additional process simulation can provide further information about the optimal strategy. With the developed toolset it is possible to design different process alternatives, which can be tested in a process simulation environment [58, 59].

## Conclusion

Given the economic and demographic trends, the topic of process optimization is ubiquitous across all healthcare. In this study the freely available s.w.an Suite toolset for detailed recording of surgical procedures was utilized and applied to arthroscopic interventions. During the data acquisition, clearly defined phases, actions, and events were recorded. In addition to recording the standard processes, a special focus was on influences that affect the perioperative process flow. Based on the data obtained, it was possible to analyze the processes before, during, and after arthroscopic surgery and generate optimization approaches. The differences between the two clinics involved, with regard to various aspects such as the spatial equipment, the personnel composition, and the partly different working methods have a visible influence on the processes and must be taken into account when evaluating the results. The contrasts between an academic teaching hospital and maximum care clinic and a highly specialized clinic enabled an interesting comparison, which reveals numerous optimization approaches.

Overall, the developed arthroscopic tool set has proven to be effective for the acquisition, analysis, decomposition, and comparison of arthroscopic workflows. The acquired SPMs provide an excellent basis for evaluating similarities and differences between the two hospitals based on detailed data. Based on this data, optimization approaches for streamlining arthroscopic surgeries have been formulated. Additionally, the arthroscopic toolset enables the analysis of different treatment approaches, surgical strategies, and skill evaluation with conformance and performance analysis. Also, the use of medical technology compared to the standard procedure can be assessed with the arthroscopic toolset in future applications.

Subsequently, the resulting SPMs can be executed in simulation scenarios and assessed based on defined key performance indicators to redesign existing processes or determine the best process alternative regarding the desired optimization goal. In this context, the implementation of the optimization approaches at the UH is the next step to show that the analysis of workflows may have a great benefit in practical everyday life. The more efficient and safer design of processes, such as in surgical procedures, will continue to be of great interest in the future. Technical progress will significantly support arthroscopic procedures as well as all other operations.

### Supplementary Information

**Additional file 1.**

## Data Availability

The datasets used and/or analyzed during the current study are available from the corresponding author on reasonable request.

## Footnotes

1 s.w.an Suite Arthroscopy Toolset, freely available at http://workflow.community
