# Supplementary material for: A comparison between a maximum care university hospital and an outpatient clinic – potential for optimization in arthroscopic workflows?
Source: BMC Health Serv Res. 2023 Nov 28;23:1313. doi: 10.1186/s12913-023-10259-3 (PMC10685488; doi:10.1186/s12913-023-10259-3)
Supplement: Supplementary file 1 — Additional file 1. [file 12913_2023_10259_MOESM1_ESM.pdf]

Supplementary Material

A. Average duration of phases and actions and average number of occurrences per intervention

Table 1 - Average duration of phases and actions and average number of occurrences per intervention

	UH	OC	*p* values
**Phases (min)**			
Preoperative	30,79 ± 9,19	26,02 ± 13,99	*p*<0,05
Postoperative	15,07 ± 9,93	9,93 ± 3,71	*p*<0,001
Changeover time	32,55 ± 7,58	6,03 ± 8,05	*p*<0,001
Closure-to-Incision-Time	80,03 ± 11,73	41,20 ± 18,79	*p*<0,001
Anaesthesia-Ready-to-Incision-Time	31,58 ± 9,74	18,03 ± 8,44	*p*<0,001
**Actions (min)**			
Positioning Patient	9,40 ± 5,60	2,73 ± 2,24	*p*<0,001
**Actions (sec)**			
Providing inside OR (per intervention)	124,52 ± 117,35	20,61 ± 30,50	*p*<0,001
Providing outside OR (per intervention)	194,86 ± 216,22	15,83 ± 36,96	*p*<0,001
Providing inside OR (per action)	25,48 ± 38,98	22,83 ± 14,03	*p*=0,6
Providing outside OR (per action)	64,54 ± 61,10	40,19 ± 32,28	*p*<0,05
**Ocurrencces (n)**			
Additional tools	5.45 ± 4.20	1.18 ± 1.48	*p*<0,001
Defective tools	0.98 ± 1.09	0.11 ± 0.31	*p*<0,001
Opening instrument set	0.28 ± 0.68	0.02 ± 0.21	*p*<0,001
Provisions inside OR	4.89 ± 3.75	0.91 ± 1.48	*p*<0,001
Provisions outside OR	3.02 ± 2.46	0.39 ± 0.67	*p*<0,001
Calls total	4.53 ± 3.44	0.11 ± 0.31	*p*<0,001
Calls surgeon	1.66 ± 1.85	0.05 ± 0.27	p<0,001
Calls surgeon sterile coated	0.25 ± 0,55	0.02 ± 0,12	*p*<0,005

B. gSPM of anterior cruciate ligament reconstruction at UH

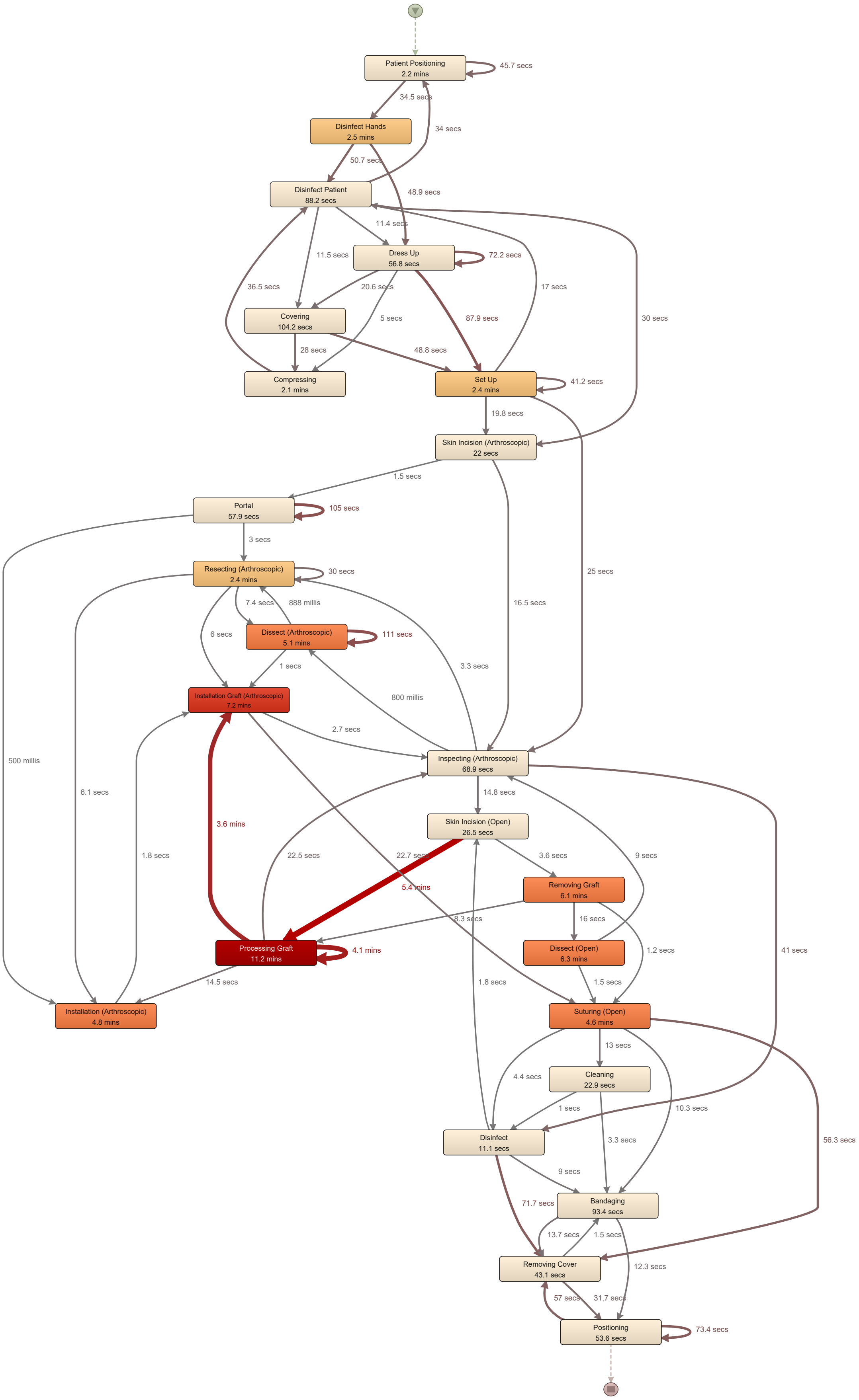

C. **gSPM of anterior cruciate ligament reconstruction at OC**

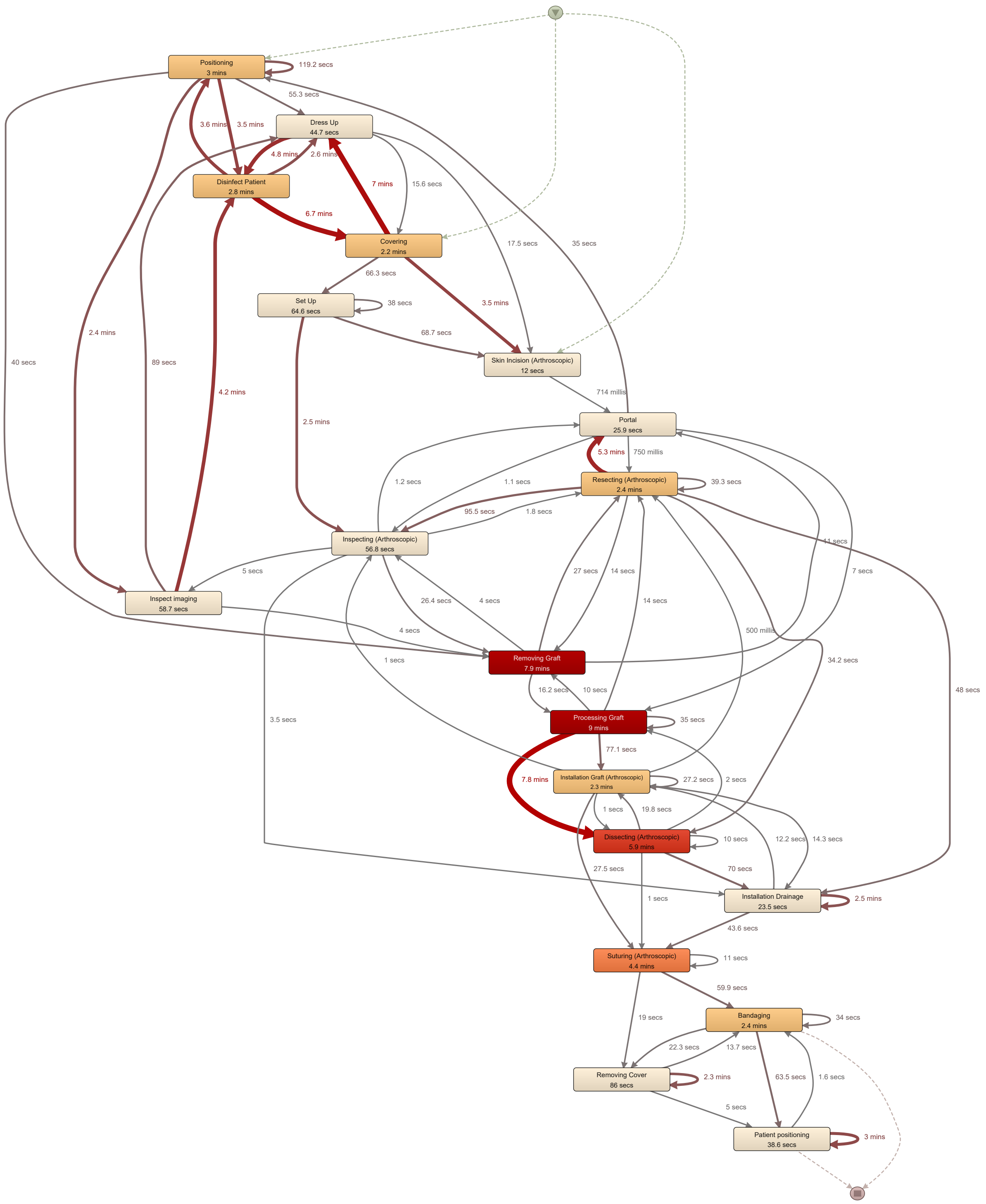

D. Definition of phases and actions

Phase	Start	End
Preoperative	Patient arrives in the operating theatre before the intervention.	Skin incision.
Postoperative	End of the last suture.	Patient leaves the operating theatre after the intervention.
Changeover time	Patient of the accomplished intervention leaves the OR.	Patient of the following intervention arrives in the OR before the intervention.
Closure-to-Incision-Time	End of the last suture of the accomplished intervention.	Skin incision in the following intervention.
Anesthesia-Ready-to-Incision-Time	Anesthetic preparation is complete and the patient is released.	Skin incision.
Intraoperative (Incision-to-Closure time)	Skin incision.	End of last suture.

Action	Start	End
Positioning Patient	First sub action to position the patient (often starting with the positioning of the patient on the operation table).	End of the last sub action to position the patient (fixation of the position).
Providing inside OR	The assistant moves to provide an instrument from inside of the OR.	The instrument is handed over to the sterile assistant.
Providing outside OR	The assistant moves to provide an instrument from outside of OR.	The instrument is handed over to the sterile assistant.
Skin incision	The scalpel cuts through the skin.	The scalpel is removed from the skin.
Suturing	Starts with the first stitch of a needle.	Ends with the last cut of the suture.
Wound dressing	Put on the wound dressing material.	Fixation of the wound dressing.
